# LNDriver: identifying driver genes by integrating mutation and expression data based on gene-gene interaction network

**DOI:** 10.1186/s12859-016-1332-y

**Published:** 2016-12-23

**Authors:** Pi-Jing Wei, Di Zhang, Junfeng Xia, Chun-Hou Zheng

**Affiliations:** 1 College of Electrical Engineering and Automation, Anhui University, Hefei, Anhui 230601 China; 2 College of Computer Science and Technology, Anhui University, Hefei, Anhui 230601 China; 3 Institute of Health Sciences, Anhui University, Hefei, Anhui 230601 China

**Keywords:** Cancer, Driver genes, Mutation data, Expression data, Interaction network

## Abstract

**Background:**

Cancer is a complex disease which is characterized by the accumulation of genetic alterations during the patient’s lifetime. With the development of the next-generation sequencing technology, multiple omics data, such as cancer genomic, epigenomic and transcriptomic data etc., can be measured from each individual. Correspondingly, one of the key challenges is to pinpoint functional driver mutations or pathways, which contributes to tumorigenesis, from millions of functional neutral passenger mutations.

**Results:**

In this paper, in order to identify driver genes effectively, we applied a generalized additive model to mutation profiles to filter genes with long length and constructed a new gene-gene interaction network. Then we integrated the mutation data and expression data into the gene-gene interaction network. Lastly, greedy algorithm was used to prioritize candidate driver genes from the integrated data. We named the proposed method Length-Net-Driver (LNDriver).

**Conclusions:**

Experiments on three TCGA datasets, i.e., head and neck squamous cell carcinoma, kidney renal clear cell carcinoma and thyroid carcinoma, demonstrated that the proposed method was effective. Also, it can identify not only frequently mutated drivers, but also rare candidate driver genes.

**Electronic supplementary material:**

The online version of this article (doi:10.1186/s12859-016-1332-y) contains supplementary material, which is available to authorized users.

## Background

Cancer is driven by genetic alterations, including single nucleotide variants (SNVs), small insertions or deletions, large copy-number variations (CNVs) and structural aberrations that accumulate during the lifetime. Several international large scale cancer genomics projects, such as The Cancer Genome Atlas (TCGA), and International Cancer Genome Consortium (ICGC) [1], etc., have produced a large volume of data in recent years [2] and provided us with an unprecedented opportunity to better characterize the molecular signatures of human cancers [3]. However, it is still a challenge to integrate information across the different omics data [4] and distinguish driver mutations which can promote the cancer cell to proliferate infinitely and diffuse from passenger mutations whose changes represent neutral variation that does not influence cancer development [5–9].

In response to the large volume of mutations being generated from massively parallel sequencing projects, many growing mathematical and statistical approaches to search for driver genes, driver pathways or core modules based on data integration were proposed. The most basic approach, eg. MutSig [10] and MuSic [11], is to identify driver genes based on somatic mutation rates in cancer patient populations, that is, the most commonly occurring mutations are more likely to be drivers [12, 13].

Also, computational approaches based on evaluating the functional impact of mutations [14] such as PolyPhen-2 [15] and OncodriverFM [16] were proposed. However, cancer is more closely related with a group of genes interacting together in a gene-gene interaction network. With the advent of the whole-genome measurements of somatic mutations and CNVs in the mass of cancer samples, many changes altered at network and pathway levels are found, not simply a point mutation [14]. Therefore, network- and pathway-based approaches have become one of the most promising methods to prioritize driver mutations and significantly mutated genes due to their abilities to model gene-gene interactions. VarWalker is a network-assisted method to prioritize potential driver genes [17]. Another method, DawnRank prioritizes altered genes on a single patient level using PageRank algorithm [3]. DriverNet is an integrated analysis framework to identify likely driver mutations by virtue of their effect on mRNA expression networks and reveals the prevalence of rare candidate driver mutations [18]. It has been demonstrated that genes which are relatively long compared to the distribution of all human consensus coding sequences (CCDS) are more likely to mutate while they may be not driver genes [17]. However, DriverNet doesn’t consider the effect of gene length. Also, the scale of the network in DriverNet is a little small which may miss some genes and the information between genes.

In this work, we develop a network-based method called Length-Net-Driver (LNDriver) to improve the performance of detecting driver genes based on the rationale of DriverNet [18]. Our goal is to consider the point mutation genes’ length and construct a new interaction network contained more genes and interactions based on Human Protein Reference Database (HPRD) [19] instead of its original gene influence graph in DriverNet. Furthermore, we integrate somatic SNV data, CNVs data and gene expression data using gene-gene interaction network. Then a greedy algorithm is applied to the integrated data to prioritize candidate genes. The application on three TCGA datasets demonstrated that the performance of our method is good.

## Methods

### The overview of LNDriver approach

**Fig. 1:**
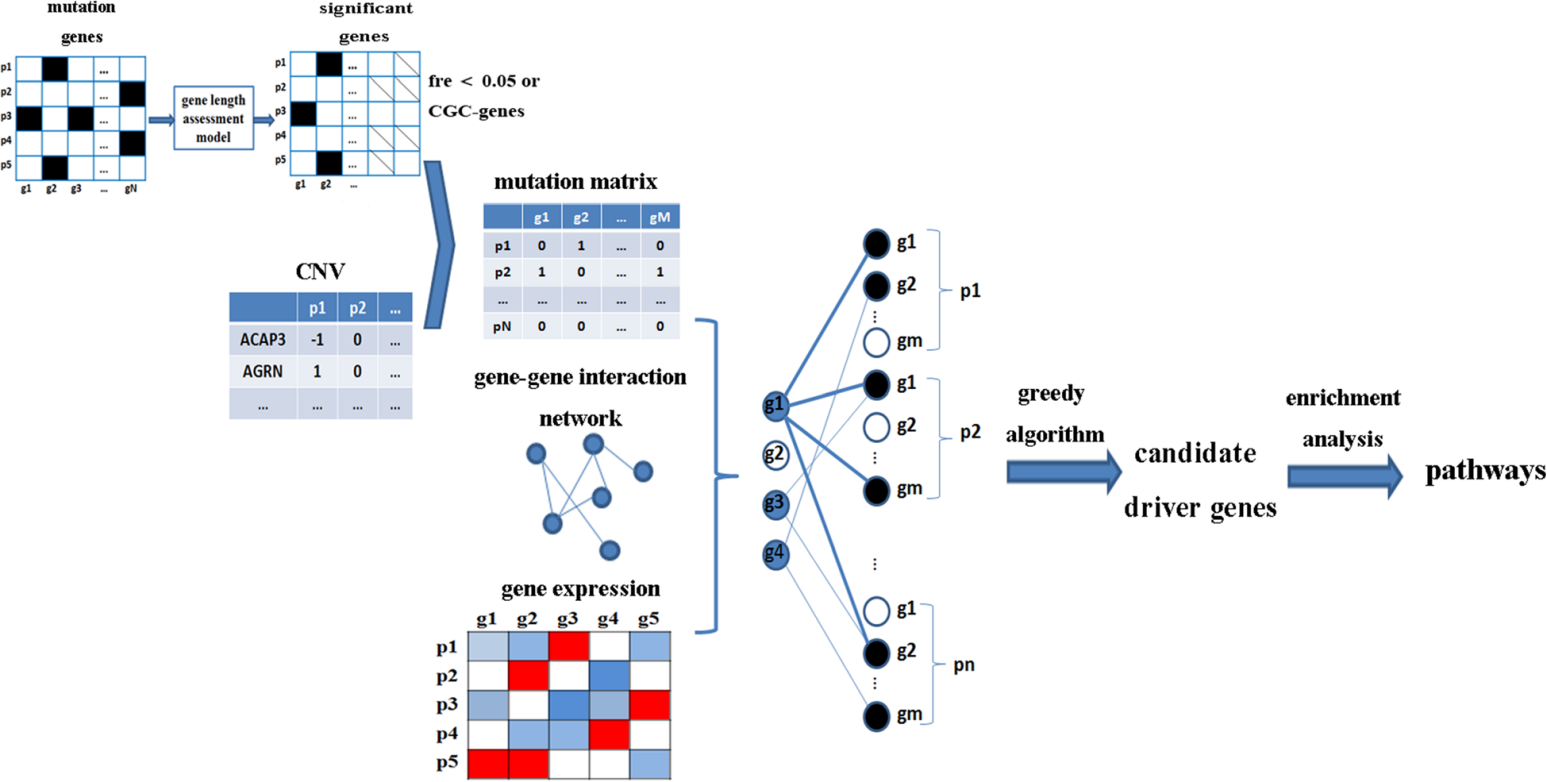
Schematic of the LNDriver. Genes in somatic mutations are firstly applied to GAM to filter long genes and then they will combine with CNV to construct mutation matrix. The bipartite graph is constructed based on mutation data, expression data and gene-gene influence network, where the blue nodes on the left bipartite graph represent the mutated gene and the black nodes on the right represent the outlying patient-gene events from the gene expression matrix. Then greedy algorithm is applied to identify candidate driver genes. Finally, enrichment analysis is employed to these candidates to explore their roles in pathways

In LNDriver method, the population-based genomic and transcriptomic interrogations of tumor types were integrated to identify driver mutations. The pipeline is shown in Fig. 1.

Actually, some studies have indicated that genes with long length have a better chance to harbor mutations (e.g. gene *TTN*) [17]. It indicated that gene length-based filtering process is essential to perform. Hence, in this study, the generalized additive model (GAM) was used to assign the somatic mutation probabilities of all human genes for each sample. Then a resampling test was performed to filter passenger genes whose occurring frequencies are ≥ 5% at random datasets [17]. After the filtering procedure, CNVs are combined with it to construct a binary mutation matrix. In addition, in order to enrich the information of the gene-gene interaction network, we constructed a new interaction network using Human Protein Reference Database (HPRD) [19]. As for gene expression data, we built a binary outlying matrix by nominating genes whose expression values are outside two standard deviation of the Gaussian distribution as outliers [18]. Next, we formulated associations between mutation and gene expression data using a bipartite graph where the left partition of nodes represented the mutation status and the right partition of nodes represented the outlying status in each of patients. After the above process, greedy algorithm was applied on the bipartite graph to select those genes in the left partition which have the highest number of outlying expression events, and then nominated them as putative driver genes. Also, the statistical significance test was assessed using a randomization framework. Finally, pathway enrichment analysis was done using the database for annotation, visualization and integrated discovery (DAVID) online tools [20, 21].

To demonstrate the advantages of the approach, we analyzed three large-scale publicly available genome-transcriptome datasets in head and neck squamous cell carcinoma (HNSC), thyroid carcinoma (THCA) and kidney renal clear cell carcinoma (KIRC).

### Filtering long genes

The length of genes in human are very different and so the mutation probabilities of different genes are in vast difference. There may be some genes which have mutations only because they are long yet they aren’t driver mutations. So, for each gene, we adopted the filtering strategies of VarWalker and computed a probability weight vector (PWV) by fitting a generalized additive model for each sample [17]. Denoting the vector *X* as the gene length of cDNA, we can adopt the following model to assess the mutated probability of a gene according to its cDNA length,

1 $$ logit\left(\pi \right)= log\left(\frac{\pi }{1-\pi}\right)\sim f(X) $$

where $\pi =\frac{\# Mutant\  Genes}{\# CCDS\  genes}$ represents the proportion of mutant genes (defined as genes with ≥ 1 deleterious somatic mutation in coding regions) in the researched samples, and *f*(⋅) represents an unspecified smooth function [17]. After the above fitting process, each gene was assigned a weight value which would be used to select genes in the next resampling procedure.

Then a resampling test was applied to random gene sets for each sample. The number of being selected random gene sets is same with mutant genes in specific sample. And the probability of each gene to be selected is based on the probability weight calculated in the above fitting procedure. The test was performed 1000 times in each sample following PWV. The mutation frequency was calculated for each gene using formula (2):

2 $$ fre=\frac{\#\left( selecting\  the\  gene\  in\  resampling\  test\right)}{1000} $$

where # (*selecting the gene in resampling test*) indicates the times and *fre* represents the frequency of the gene being selected across 1000 times in resampling process. Then we filtered those genes whose frequencies were ≥ 5% that indicates the gene may occur at random unless they are CGC genes. Those genes with *fre* < 5% which represented the gene was unlikely mutated at random were observed.

### Greedy algorithm

For detecting the candidate driver genes based on processed mutation data and expression data, they were integrated with the gene-gene interaction network into a bipartite graph (see Fig. 1). The elements on the left of bipartite graph represent the mutation status of genes in population level. And the right partition events indicate outlying expression status of the genes [18]. An edge between *g*_*i*_ and *g*_*j*_ will be drawn if the gene *g*_*i*_ in the left partition is mutated (blue node), the right gene *g*_*j*_ is outlying expression gene (black node) and *g*_*i*_ interacts with *g*_*j*_ in the gene-gene interaction network. Given the bipartite framework, the aim is to find the mutation genes on the left partition which cover the most events on the right of bipartite graph. To this end, the optimization method of a greedy algorithm was used to select the most covered genes: at each step, chose a mutated gene which connected to the most uncovered outlying expression genes on the right of bipartite graph. When all the connected outlying expression events were covered, the program was terminated. Finally, the mutated genes ranked based on their coverage and the mostly covered mutated genes are considered as the candidate driver genes.

### Significance test

In order to assess the statistical significance of the candidate driver genes, the random framework was used by permuting *N* = 100 times of the original datasets including mutation matrix, processed outlying expression matrix and the gene-gene interaction network. Then the algorithm was run on the *N* randomly generated datasets. Finally, the real data results were assessed to see whether they are significantly different from the results on randomized datasets. The null hypothesis *H*_0_ is that the gene mutations have no influence on the occurrence of the cancer, and the alternative hypothesis *H*_1_ is that the cancer is related to the mutations of the genes. The definition of the statistical significance of gene *g*, whose corresponding node coverage is *COV*_*g*_, is the fraction times of selecting driver genes that are more than *COV*_*g*_ in *N* = 100 random runs of the method. The calculation is listed as follows:

3 $$ p- value(g)=\frac{{\displaystyle {\sum}_{i=1}^N}{\displaystyle {\sum}_{j=1}^{Si}}\delta \left[CO{V}_{gij}>CO{V}_g\right]}{{\displaystyle {\sum}_{i=1}^N}{S}_i} $$

where *S*_*i*_ is the number of candidate driver genes selected in the *i*th run of the method [18]. Then the Benjamini-Hochberg method was used to correct the *p*-*values* for multiple tests and finally we chose the genes whose *p*-*values* were less than 0.05.

## Results

### Datasets and pre-processing

We applied LNDriver to 513 THCA samples, 522 HNSC samples and 534 KIRC samples (Table 1). These three datasets comprise somatic SNV data, CNV data and gene expression data collected from The Cancer Genome Atlas (TCGA) data portal [22].

**Table 1:** Description of datasets

Tumor type	Number of tumor expression samples	Number of somatic mutation samples	Samples of tumor expression∩somatic samples
THCA	513	435	433
HNSC	522	509	501
KIRC	534	417	415

### The construction of mutation matrix

Firstly, we collected somatic SNVs in level 2 and CNV data in level 3 directly from TCGA data portal. Secondly, we removed the genes whose item of “Variant_Classification” is “silent” or “RNA” in somatic SNV data and whose length are too long according to generalized additive model and resampling test process. Thirdly, the CNV information was extracted by selecting genes from amplified and deleted segments in CNV data. Finally, we integrated CNV data with filtered somatic SNV data by getting intersecting samples and union genes to construct a binary matrix *M*, whose rows indicate samples and columns indicate genes. Each entry of *M*_*ij*_ refers to the mutation status of gene *j* in sample *i* and *M*_*ij*_ = 1 represents that there is labeled valid mutation in gene *j* of sample *i*. Otherwise, *M*_*ij*_ = 0 indicates the absence of a mutation in the *j*th gene of the *i*th sample.

### Expression outlier matrix

For gene expression dataset *E*, the values of it contain not available (*NA*) values. These values affect the results of the approach. We substituted them with the mean of all other genes in the specific samples. Also, we adopted the assumption in DriverNet that the expression distribution of every gene across all samples is Gaussian distribution [23]. Based on the hypothesis, we converted the expression data to a binary patient-outlier matrix *E* ' where *E* ^'^(*i*, *j*) = 1 means the expression of gene *i* is an outlier in patient *j*. The definition of the outliers is that genes whose expression values are outside the two-standard deviation range of the expression values of gene *i* across all the patients [18].

### Gene-gene interaction network and gene annotation data

Cancer is a disease related with sets of genes which interact with each other in some molecular networks not only related with single gene. In order to enrich the information gene-gene interaction network in DriverNet, we built an influence graph *G*(*V*, *E*) using HPRD [19] (release 9, 06/29/2010) which contains 9617 proteins to server as our reference network. The influence graph *G*(*V*, *E*) in our work is an undirected and unweighted binary network where *V* represents the nodes of genes and *E* represents the edges among genes. When there is a correlation between gene *i* and gene *j*, *G*_*ij*_ = 1, otherwise *G*_*ij*_ = 0.

We used the consensus coding sequences (CCDS) genes data which have been allocated complementary DNA (cDNA) length based on their coding sequences from VarWalker [17] as a benchmark gene resource to select those genes that have matched CCDS symbols. In order to explore the impact of the gene length, we compared genes with somatic SNVs with the distribution of all human CCDS gene length to filter long genes.

### Cancer gene census (CGC) genes

The CGC is a database that catalogues genes whose mutations have been causally implicated in cancer, which has been widely served as benchmark in many cancer researches. In this work, we also utilized it as the standard reference list which was downloaded from COSMIC [24] and included total of 571 genes (07/8/2015).

### The analysis of the overall performance

In this study, the performance of LNDriver’s ability was evaluated using the number of indentifying known drivers in CGC database compared with other methods. The benchmarks of the above evaluation were precision, recall and F1score which were based on the top *N* genes as following:

4 $$ precision=\frac{\left(\# Mutated\kern0.5em  genes\kern0.5em  in\kern0.5em CGC\right)\cap \left(\# Genes\kern0.5em  found\kern0.5em  in\kern0.5em  LNDrivers\right)}{\left(\# Genes\kern0.5em  found\kern0.5em  in\kern0.5em  LNDrivers\right)} $$

5 $$ recall=\frac{\left(\# Mutated\kern0.5em  genes\kern0.5em  in\kern0.5em CGC\right)\cap \left(\# Genes\kern0.5em  found\kern0.5em  in\kern0.5em  LNDrivers\right)}{\left(\# Mutated\kern0.5em  genes\kern0.5em  in\kern0.5em CGC\right)} $$

6 $$ F1\kern0.5em  score=2\times \frac{precision\times recall}{precision+ recall} $$

**Fig. 2:**
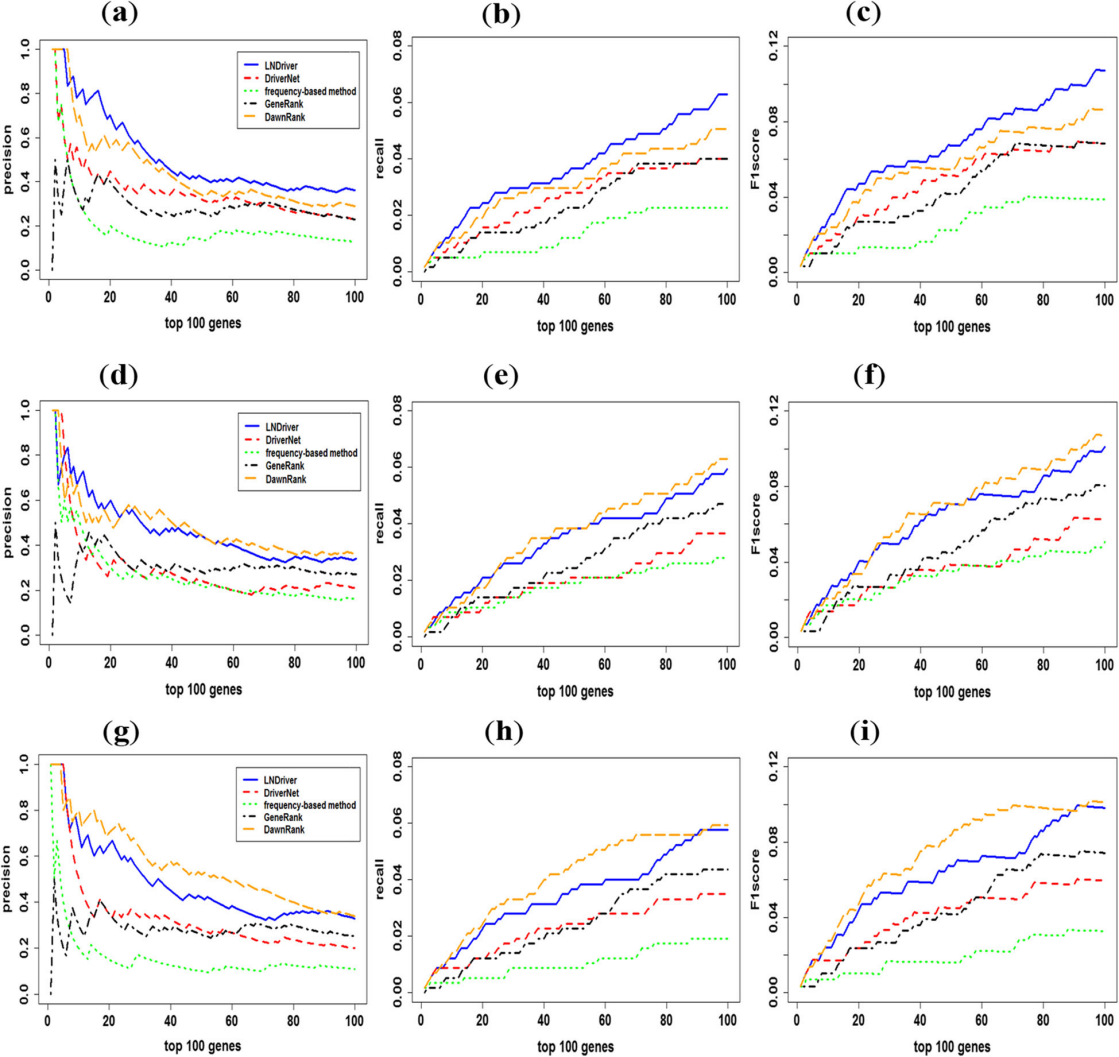
**a** HNSC precision. **b** HNSC recall. **c** HNSC F1score. **d** KIRC precision. **e** KIRC recall. **f** KIRC F1score. **g** THCA precision. **h** THCA recall. **i** THCA F1score. The comparison of precision, recall and F1score for top ranking genes in LNDriver and other methods. The *X* axis represents the number of top ranking genes and the *Y* axis represents the score of the precision, recall and F1score respectively

For the sake of performing the property of our method on identifying cancer related drivers, we compared the result of our method to classical frequency-based method, GeneRank method [25], DriverNet method and personal-based method of DawnRank. The results of the experiment on HNSC, KIRC and THCA datasets are shown in Fig. 2.

HNSC, the sixth most common cancer worldwide [26], was analyzed in our method. As for the overall performance of its top 100 genes, it can be seen in Fig. 2a-c that LNDriver method remarkably outperforms other four methods. For the top 100 genes, there are 36 genes contained in CGC database of our method, while 32 of DawnRank and 23 of DriverNet. There are 200 genes being selected as candidates and 32 genes of them with *p-values* less than 0.05 in our method (see Additional file 1). Apart from those common genes like *TP53*, *EGFR, CDKN2A* and *PIK3CA,* the *NOTCH1* which functioned as tumor suppressor gene in HNSC was also indentified in our method [26]. In addition, *CASP8*, which is ranked 16 in our method while 58 in DriverNet, has been demonstrated that in human papillomavirus (−) HNSC, concurrent mutations of *CASP8* with *HRAS* can target cell cycle, death, *NF-κB* and other oncogenic pathways [27]. Furthermore, *PPFIA1* gene, which was ranked 9 in our method while was not detected in DriverNet, acts as an invasion inhibitor in HNSC and is the highest upregulated gene in the 11q13 amplicon of HNSC cell lines [28].

For KIRC data set, our method always remarkably outperforms GeneRank and frequency-based method (Fig. 2d-f). Although the performance of the top several genes in LNDriver is slightly worse than DriverNet and DawnRank, for latter genes, it has a remarkably better performance than DriverNet method. The curves show that the stability of our method and DawnRank is relatively good since the precision of the two methods are similar. About top 100 genes, 34 are found in CGC in our method. In LNDriver, 164 genes are indentified as candidates and 36 of them with *p* − *value* ≤ 5% (see Additional file 2). Indeed, some well validated genes such as *VHL*, *TP53*, *EGFR*, *PTEN* and so on are ranked in the top rank in our method. Interestingly, *EWSR1* (also known as *EWS*) in CGC is not nominated as candidate drivers in DriverNet and DawnRank, while it is one of the most commonly involved genes in sarcoma translocations [29].

For THCA, although the performances of LNDriver on top several genes are same with DriverNet, the overall effect is better than DriverNet, frequency-based, and GeneRank method (Fig. 2g-i). In middle part of the top 100 genes (from the 6th gene to about 90th gene), our method performs poor than DawnRank in this dataset, but the top 5 genes are all in CGC. After the significance test, we chose 34 genes whose *p-values* were less than 0.05 as the cancer driver genes (see Additional file 3). With respect to several top genes, like *PTPN11*, it encodes the protein-tyrosine phosphatase *SHP2* whose protein expression was significantly increased in human thyroid carcinoma [30]. In addition, there are literatures suggesting that somatic gain-of-function mutations of *PTPN11* are presented in breast cancer [30, 31], lung adenocarcinomas [32] and etc. *BRAF* is ranked as the second impactful driver gene which is an important event in the development of papillary thyroid cancer [33]. For the *RAS* genes (*HRAS* and *NRAS*), upon activation they can activate the *MAPK* pathway [34] which plays an essential role in the control of the cell cycle and differentiation [35].

### The analysis of identifying rare drivers

LNDriver can identify not only frequently mutated driver genes, but also rare significant drivers. The ‘rare significant drivers’ are defined as genes with *p* − *values* < 0.05 and whose alteration frequencies are less than 2% of the patient cohort in mutation data.

In HNSC, we obtained 8 rare genes (see in Table 2) in 32 candidate drivers with *p* − *values* < 0.05. Four of them (*AKT1, RB1, PLCG1, ZBTB16*) are in CGC. For example, *AKT1* (1.99% of cases), identified by LNDriver, is a serine/threonine protein kinase and its downstream proteins have been reported to be frequently activated in human cancers [36]. The *RB1* gene is tumor suppressor gene identified and loss of it is considered an accelerating event in retinoblastoma [37, 38].

**Table 2:** The rare driver genes in HNSC

Rank	Gene	Cases with mutations	Mutation frequency (%)	*p*-value	CGC gene
14	*AKT1*	10	1.996008	0.011832	YES
15	*RB1*	9	1.796407	0.012938	YES
18	*CALM1*	7	1.397206	0.016769	NO
22	*MAPK1*	4	0.798403	0.019237	NO
23	*PLCG1*	5	0.998004	0.030388	YES
24	*ZBTB16*	8	1.596806	0.032729	YES
30	*SETDB1*	3	0.598802	0.044476	NO
32	*PTK2*	4	0.798403	0.048264	NO

For KIRC, 29 rare drivers were identified in our method and 11 of which are in CGC (see in Table 3). Although some rare genes like *EGFR, EP300* and *CREBBP* are found in DriverNet, but the ranked positions are more near to the top in our method. In addition, the activity of *SRC* (0.48% of cases), although it isn’t contained in CGC, is often associated with disease and might contribute to the development of human malignancy [39]. The *Src* family of protein tyrosine kinases provides us with many important landmarks in understanding oncogenic transformation [39]. Furthermore, *CDKN2A* (1.20% of cases) and *RB1* (1.03% of cases) are hallmarks of lung squamous cell carcinoma [40] and glioblastoma [41] respectively.

**Table 3:** The rare driver genes in KIRC

Rank	Gene	Cases with mutations	Mutation frequency (%)	*p*-value	CGC genes
3	*SRC*	2	0.481928	0.001378	NO
5	*EGFR*	7	1.686747	0.003100	YES
6	*EP300*	6	1.445783	0.003214	YES
7	*CHD3*	4	0.963855	0.004018	NO
8	*EWSR1*	2	0.481928	0.00551	YES
9	*ATF7IP*	5	1.204819	0.007462	NO
11	*RB1*	1	0.240964	0.010332	YES
12	*NCOA3*	5	1.204819	0.011135	NO
13	*PRKCD*	2	0.481928	0.011135	NO
14	*CREBBP*	4	0.963855	0.012513	YES
15	*DDX20*	4	0.963855	0.012513	NO
16	*SMAD9*	1	0.240964	0.013546	NO
17	*KDR*	5	1.204819	0.016186	YES
19	*PPARG*	1	0.240964	0.018138	YES
21	*ATXN1*	2	0.481928	0.021008	NO
22	*HDAC1*	2	0.481928	0.021008	NO
23	*PLG*	5	1.204819	0.021008	NO
24	*CDKN2A*	5	1.204819	0.023533	YES
25	*MET*	3	0.722892	0.023533	YES
26	*EIF6*	1	0.240964	0.027322	NO
27	*JAK2*	5	1.204819	0.027322	YES
29	*PCNA*	3	0.722892	0.032717	NO
30	*ARF6*	1	0.240964	0.039031	NO
31	*FRS2*	2	0.481928	0.039031	NO
32	*SETDB1*	4	0.963855	0.039031	NO
33	*NOS1*	8	1.927711	0.044886	NO
34	*PPP2R1A*	2	0.481928	0.044886	YES
35	*RAB5A*	1	0.240964	0.044886	NO
36	*SVIL*	7	1.686747	0.044886	NO

For THCA, in addition to the frequently mutated genes (*PTPN11, BRAF, HRAS, NRAS and CDC27*), the rest of the drivers indentified by our method are rare genes (Table 4). For example, *PTK2B* is a member in *PAK* signaling pathway [42].

**Table 4:** The rare driver genes in THCA

Rank	Gene	Cases with mutations	Mutation frequency (%)	*p*-value	CGC genes
3	*RB1*	6	1.385681	0.000101	YES
4	*TP53*	3	0.692841	0.000101	YES
6	*PRKACA*	2	0.461894	0.002121	NO
7	*PTK2B*	2	0.461894	0.004141	NO
8	*PIK3R1*	2	0.461894	0.005858	YES
9	*EP300*	3	0.692841	0.006868	YES
10	*PTPN6*	1	0.230947	0.008484	NO
11	*CASP3*	1	0.230947	0.009191	NO
12	*JAK2*	2	0.461894	0.009191	YES
14	*YWHAG*	1	0.230947	0.009191	NO
15	*CDKN1A*	1	0.230947	0.009696	NO
16	*PTEN*	6	1.385681	0.010706	YES
17	*CTNNB1*	4	0.923788	0.018079	YES
18	*ACTB*	1	0.230947	0.020099	NO
19	*PML*	8	1.847575	0.020099	YES
20	*ATM*	5	1.154734	0.022725	YES
21	*HSP90AA1*	1	0.230947	0.022725	YES
22	*SMAD3*	1	0.230947	0.026462	NO
24	*FLNC*	5	1.154734	0.035754	NO
25	*BRCA1*	6	1.385681	0.041713	YES
26	*CHD3*	4	0.923788	0.041713	NO
27	*CHEK2*	7	1.616628	0.041713	YES
28	*GRIN2B*	5	1.154734	0.041713	NO
29	*NEDD4*	5	1.154734	0.041713	NO
30	*PIAS4*	2	0.461894	0.041713	NO
31	*RASA1*	2	0.461894	0.041713	NO
32	*VAV1*	1	0.230947	0.041713	NO
33	*ACTA1*	1	0.230947	0.048783	NO
34	*SP1*	1	0.230947	0.048783	NO

### Long genes filtering analysis

In this study, we adopted GAM to assign every point mutation gene with a probability weight consequently to filter frequent mutations because of long length. With respect to *TTN* gene, the longest gene in human, ranked 18 as a driver gene of HNSC by DriverNet algorithm. However, after the step of filtering long genes in our improved method, it just ranked 140 and wasn’t nominated as a candidate of driver gene. And in THCA, our method didn’t identify *TTN* as a candidate while it was detected as the fourth ranked gene in frequency-based method.

### Enrichment analysis

To test biological functions of these predicted candidate drivers, KEGG pathway enrichment and GO functional enrichment were performed using DAVID tool (v6.8).

For HNSC, the important candidates are mainly enriched in pathways in cancer, prostate cancer, glioma, non-small cell lung cancer, melanoma, ErbB signaling pathway and so on after KEGG pathway enrichment (see Additional file 4). With respect to the biological process, regulation of apoptosis, programmed cell death, cell death, nitrogen compound metabolic process, cellular biosynthetic process and etc. are enriched after the GO functional enrichment (see Additional file 4). Concerning the cellular component, identified candidates are enriched in nuclear lumen, nucleoplasm, intracellular organelle lumen, organelle lumen, membrane-enclosed lumen and cytosol etc*.* (see Additional file 4)*.* Furthermore, with regard to important molecular functions, candidate drivers are enriched in identical protein binding, nitric-oxide synthase regulator activity, structure-specific DNA binding, transcription factor binding, enzyme binding and so on (see Additional file 4).

In KIRC, pathways in cancer, cell cycle, melanoma and prostate cancer etc. are enriched in KEGG pathways (see Additional file 5). In terms of biological process, positive regulation of nitrogen compound metabolic process, cellular biosynthetic process, biosynthetic process, cell cycle, transcription and gene expression etc. are significantly enriched in GO functional enrichment (see Additional file 5). As for cellular component, candidates are enriched in nucleoplasm, nuclear lumen, nucleoplasm part, nuclear periphery, chromosome and so on (see Additional file 5). In terms of molecular functions, transcription factor binding, protein tyrosine kinase activity, transcription regulator activity and nucleotide binding etc*.* are enriched (see Additional file 5).

In THCA, the pathways after KEGG enrichment are prostate cancer, pathways in cancer, chronic myeloid leukemia and glioma etc*.* (see Additional file 6). In terms of biological process in GO functional enrichment, candidate drivers are enriched in response to organic substance, apoptosis, programmed cell death and induction of apoptosis by intracellular signals etc*.* (see Additional file 6). With respect to cellular component, cytosol, nucleoplasm, nuclear lumen, intracellular organelle lumen and so on are enriched (see Additional file 6). As for molecular functions, candidates are enriched in enzyme binding, enzyme binding, protein serine/threonine kinase inhibitor activity and protein kinase binding etc. (see Additional file 5).

## Discussion and conclusions

In this work, we introduced a network-based framework by integrating transcriptome and genomics data into a gene-gene interaction network to identify significant driver gene in cancer. By virtue of the consideration of gene length, the frequently mutated genes with long length may be filtered. Also, we constructed a network containing more genes and interaction information in order to improve the accuracy of driver genes identifying. LNDriver can identify not only frequently mutations but also rare drivers. Application on HNSC, KIRC and THCA datasets has demonstrated that the performance of our method is remarkably better than frequency-based, GeneRank and DriverNet method. In addition, our method also outperforms DawnRank method in HNSC dataset. However, in KIRC and THCA, DawnRank sometimes have a better performance than our method. We will explore the causes about this phenomenon in our following work and we hope to find a new method which can have a good performance on KIRC and THCA.

Furthermore, there are also some limitations of our method. Firstly, gene length filtering step was only applied to point mutations not including CNVs because point mutations are more inclined to be affected by gene length. Although this step has ability to filter long genes, it has randomness. We will seek solutions to improve it and enhance robustness of it. Secondly, the information of gene-gene interaction network are more and more abundant with the development of the field. So, we will try to integrate more information to a new gene-gene interaction network which may help us to mine more information about cancer driver genes. Moreover, it is now acknowledged that precision medicine and personalized medicine are important for patient diagnosis and treatment, so we will major in proposing new method to identify patient-specific and rare driver genes based on individual mutational and expression profiles in different tumors in the future.

## Additional files

Additional file 1: The candidate drivers with *p-values* less than 0.05 in HNSC. (XLS 20 kb)
Additional file 2: The candidate drivers with *p-values* less than 0.05 in KIRC. (XLS 19 kb)
Additional file 3: The candidate drivers with *p-values* less than 0.05 in THCA. (XLS 19 kb)
Additional file 4: The results of KEGG and GO enrichment analysis in HNSC. (XLS 114 kb)
Additional file 5: The results of KEGG and GO enrichment analysis in KIRC. (XLS 122 kb)
Additional file 6: The results of KEGG and GO enrichment analysis in THCA. (XLS 158 kb)

## Abbreviations

CCDS: Consensus coding sequences
cDNA: complementary DNA
CGC: Cancer gene census
CNVs: Copy-number variations
DAVID: Database for annotation, visualization and integrated discovery
GAM: Generalized additive model
HNSC: Head and neck squamous cell carcinoma
HPRD: Human protein reference database
ICGC: International cancer genome consortium
KIRC: Kidney renal clear cell carcinoma
LNDriver: Length-Net-Driver
NGS: Next-generation sequencing
PWV: Probability weight vector
SNVs: Single nucleotide variants
TCGA: The Cancer Genome Atlas
THCA: Thyroid carcinoma
